# Geriatric Nutritional Risk Index can predict postoperative delirium and hospital length of stay in elderly patients undergoing non‐cardiac surgery

**DOI:** 10.1111/ggi.13963

**Published:** 2020-06-22

**Authors:** Yanli Zhao, Xin Xia, Dongmei Xie, Yulin Liao, Yanyan Wang, Ling Chen, Ning Ge, Jirong Yue

**Affiliations:** ^1^ Department of Geriatrics and National Clinical Research Center for Geriatrics West China Hospital of Sichuan University Chengdu China; ^2^ Department of Respiratory Diseases First Affiliated Hospital of Guangxi Medical University Nanning China

**Keywords:** elderly, Geriatric Nutritional Risk Index, length of stay, postoperative delirium

## Abstract

**Aim:**

Malnutrition is common in older patients and is associated with adverse outcomes. The Geriatric Nutritional Risk Index (GNRI) has been developed as an objective index to assess nutritional risk in these patients. However, there is limited evidence regarding the association between GNRI and postoperative delirium (POD) as well as length of stay (LOS) in surgical older patients. Therefore, our goal was to examine the impact of poor nutrition, evaluated by the GNRI, on POD and length of stay in older patients undergoing non‐cardiac surgery.

**Methods:**

In this prospective cohort study, older patients undergoing non‐cardiac surgery from April to June of 2015 were included. Preoperative nutritional status was assessed by the GNRI within the first 48 h after hospital admission. The outcomes were POD and LOS. Multivariable logistic regression and linear regression analyses were used to assess the role of GNRI in predicting these outcomes.

**Results:**

In the whole sample (*N* = 288), the prevalence of severe/moderate (GNRI <92) and low (GNRI 92–98) nutritional risk was 15.6% and 29.5%, respectively. The median length of hospital stay was 14 days. Delirium was present in 49 patients (17%). A linear regression analysis showed that low (β = 4.56, 95% confidence interval [CI]: 2.18–6.94) and severe/moderate (β = 3.70, 95% CI: 0.74–6.65) nutritional risk were associated with prolonged LOS. Moreover, a multivariate analysis revealed that patients with severe/moderate nutritional risk were more likely to develop POD compared with those without (odds ratio = 2.56, 95% CI: 1.11–5.89).

**Conclusion:**

Preoperative malnutrition, as assessed using the GNRI, predicted LOS and the development of POD in elderly non‐cardiac surgical patients. **Geriatr Gerontol Int 2020; 20: 759–764**.

## Introduction

Malnutrition is a common medical comorbidity, particularly in patients >65 years of age.^1^ The prevalence of malnutrition in hospitalized patients ranges from 10% to 50% depending on the criteria used for diagnosis and the population studied.^2^ Both reduced food intake and illness‐induced alterations to patient metabolism can drive malnutrition in elderly individuals. With global increases in life expectancy and advances in medical science and technology, steadily increasing numbers of older patients are undergoing surgery. Malnutrition commonly observed in surgical elderly patients. Furthermore, this condition has been shown to be associated with morbidity, mortality and an increase of healthcare costs.^3^, ^4^ It was also shown to correlate with delirium and prolonged hospitalization.^5^, ^6^ Evidence have shown that nutritional support can reduce length of stay, morbidity, mortality and incidence of delirium.^4^, ^7^, ^8^ However, malnutrition is occasionally unrecognized and not treated properly in hospitalized patients.^3^ Therefore, it is important to identify patients at risk of malnutrition early and to treat these patients adequately.

Recently, several strategies have been proposed for the assessment of nutritional status in older adults, with the Mini Nutritional Assessment (MNA) being recommended by the European Society for Clinical Nutrition and Metabolism (ESPEN).^9^ However, this assessment is dependent on administering a questionnaire and thus cannot be used for older adults with dementia or difficulties in communicating. Faced with these problems and the difficulties in determining standing height and regular weight in older individuals, the Geriatric Nutritional Risk Index (GNRI), a specific screening index for the elderly, has been proposed.^10^ The GNRI is a simple and objective index, allowing clinicians to assess patients readily only based on height, weight and serum albumin level.^10^ To date, it has been proposed for the evaluation of at‐risk elderly hospital patients, hemodialysis patients, sepsis patients and cardiovascular patients.^11^, ^12^, ^13^, ^14^ However, few studies have investigated the predictive value of the GNRI in perioperative older patients, particularly there is limited evidence regarding the impact of GNRI on postoperative delirium (POD) and length of stay (LOS). The GNRI was developed as a novel and feasible screening tool to assess the nutritional risk for the elderly. Therefore, it is essential to apply the GNRI in surgical elderly patients.

Thus, the aim of this study was to investigate whether nutritional risk, as assessed by the GNRI, is associated with POD and LOS in elderly patients undergoing non‐cardiac surgery. We hypothesized that malnutrition screening by GNRI would be an independent predictor of POD and LOS in older non‐cardiac surgical patients.

## Methods

### *Study design and population*

This prospective cohort study was conducted in the West China Hospital, Sichuan University from April to June of 2015. Eligible patients were aged ≥70 years, scheduled to undergo non‐cardiac surgery, and had an anticipated length of stay of at least 3 days. Exclusion criteria were: (i) severe hearing impairment and inability to communicate, (ii) history of dementia or psychiatric illness, (iii) terminal condition (not expected to survive longer than 6 months), (iv) presence of delirium at admission, and (v) underwent neurologic surgery.

The study was approved by the Institutional Review Boards of West China Hospital, Sichuan University and was performed in accordance with the principles of the Declaration of Helsinki. All the participants were asked to provide written informed consent.

### *Data collection*

#### *Assessment of preoperative factors*

All patients were assessed by trained research nurses preoperatively within 48 h of admission. The demographic characteristics that were assessed included age, gender, level of education, marital status and living situation. Perioperative information was also gathered on alcohol abuse, smoking and type of surgery (orthopedic, general, thoracic). Preoperative pain was assessed using the Facial Scale (range 0–10) by patient interview.^15^ Comorbidities were evaluated using the Charlson Comorbidity Index (CCI).^16^

#### *Assessment of nutrition*

Malnutrition was evaluated using the GNRI within 48 h of admission. The GNRI, an adaptation of the Nutritional Risk Index (NRI) designed by Buzby *et al*.,^17^ is a simple nutritional screening tool used to evaluate nutrition‐related risk in surgical patients. We collected the following nutritional parameters: height, weight, body mass index and serum albumin. The index was calculated as follows:^1^ GNRI = 1.489 × serum albumin (g/L) + 41.7 × present weight/ideal weight (kg). Ideal body weight was derived using the following equations of Lorentz:^1^ ideal weight for men = 0.75 × height (cm) – 62.5, ideal weight for women = 0.60 × height (cm) – 40. Study participants were stratified into the following three categories: no risk (GNRI >98), low risk (92–98), and severe/moderate risk (GNRI <92).^1^ Unlike the original grouping into four classes proposed by Bouillanne *et al*.,^10^ we avoided distinguishing between the severe risk (GNRI *<*82) and the moderate risk (GNRI 82 to <92) groups, because these two categories have been shown to present with a similar increased risk of overall health complications.^10^

#### *Outcomes*

The primary outcome was POD. Beginning on the first postoperative day, the Confusion Assessment Method (CAM) was used by the trained research assessors to screen patients for delirium.^18^ The CAM is developed based on four criteria found in the Diagnostic and Statistical Manual of Mental Disorders, Fourth Edition, Text Revision (DSM‐IV‐TR): (i) acute onset and fluctuating course, (ii) inattention, (iii) disorganized thinking and (iv) altered level of consciousness. A positive diagnosis of delirium required the presence of items (i) and (ii), and either (iii) or (iv). The screening instrument is a widely used tool with very high sensitivity, specificity and inter‐rater reliability. The second outcome was LOS, defined as the number of days in hospital from the day of admission to the day of discharge.

### *Statistical analysis*

All statistical analyses were performed using IBM SPSS Statistics software v21.0 (IBM Corp., Armonk, NY, USA). Continuous variables were expressed as means with the standard deviation (SD) for normally distributed data and the median with the interquartile range (IQR) for non‐normally distributed data. ANOVA analyses and Kruskal–Wallis tests were used for between‐group comparisons of continuous variables with normal and non‐normal distributions, respectively. Categorized variables were expressed as the number of cases and percentages, and were compared using chi‐squared tests.

Univariate logistic regression analyses were conducted to examine the association between each potential risk factor and POD. Variables that were significantly associated with POD in the univariate analyses were then entered into the multivariable logistic regression. For the evaluation of the association between GNRI and prolonged LOS, a linear regression model was constructed. Model 1 adjusts for age and sex. Model 2 adjusts for age, sex, CCI and type of surgery. *P* < 0.05 was considered statistically significant.

## Results

In total, 348 patients were assessed, of whom 28 and 32 were excluded from the final analysis due to cancelling of the scheduled operation and incomplete data, respectively. Therefore, 288 subjects were included in the final analysis. The median age of these patients was 74 years (IQR = 6). More than half (51.4%) were men. Of these patients, 49 (17%) developed POD. The median length of hospital stay was 14 days (IQR = 11) and the mean ± SD of GNRI was 98.98 ± 8.46. The number of patients who underwent general, orthopedic and thoracic surgery were 189 (65.6%), 71 (24.7%) and 28 (9.7%), respectively. The incidence of POD for each type of surgery was as follows: 19% (36 of 189) in general surgical patients, 8.5% (six of 71) in orthopedic surgical patients and 25% (seven of 28) in thoracic surgical patients. The median number of hospital days among general, orthopedic, thoracic surgical patients were 14 (IQR = 9), 14 (IQR = 12) and 23 (IQR = 11), respectively.

Table 1 shows the characteristics of these patients according to GNRI cutoff values. The prevalence of no, low and severe/moderate nutritional risk was 51.7%, 34% and 14.2%, respectively. There were no significant differences in age, gender, alcohol abuse, smoking, type of surgery, preoperative pain or CCI among nutritional risk categories. The severe/moderate nutritional risk group had lower body mass index and serum albumin than the other groups. Incidence of POD was significantly higher in the severe/moderate risk group compared with no and low‐risk groups. In addition, low and severe/moderate risk patients had a longer average length of stay than those with no risk.

**Table 1:** Baseline characteristics of patients undergoing non‐cardiac surgery according to GNRI categories

Characteristic	Total	Severe/moderate risk (*n* = 45)	Low risk (*n* = 85)	No risk (*n* = 158)	*P*‐value^†^
Age (years), median (IQR)	74 (6)	75 (7)	75 (7)	74 (6)	0.086
Male gender, *n* (%)	148 (51.4)	24 (53.3)	36 (42.4)	88(55.7)	0.134
Living alone, *n* (%)	17 (5.9)	4 (8.9)	4 (4.7)	9 (5.7)	0.621
Married, *n* (%)	232 (80.6)	32 (71.1)	67 (78.8)	133 (84.2)	0.132
Alcohol abuse, *n* (%)	60 (20.8)	8 (17.8)	22 (25.9)	30 (19)	0.388
Smoker, *n* (%)	84 (29.2)	14 (31.1)	24 (28.2)	46 (29.1)	0.943
Preoperative pain, *n* (%)	169 (58.7)	26 (57.8)	56 (65.9)	87 (55.1)	0.261
Education, *n* (%)					
Illiteracy or primary school	139 (48.3)	26 (57.8)	42 (49.4)	71 (44.9)	
Middle school	49 (17)	8 (17.8)	16 (18.8)	25 (15.8)	0.061
High school or above	100 (34.7)	11 (24.4)	27 (31.8)	62 (39.2)	
BMI (kg/m^2^), mean ± SD	22.32±3.29	19.96±3.49^‡^	21.54±3.30^‡^	23.40±2.72^‡^	<0.001
Albumin (g/L), mean ± SD	40.12±4.87	32.87±4.14^‡^	38.06±2.31^‡^	43.29±2.86^‡^	<0.001
CCI, *n* (%)					
Mild (≤2)	214 (74.3)	34 (75.6)	58 (68.2)	122 (77.2)	
Moderate (3, 4)	52 (18.1)	7 (15.6)	21 (24.7)	24 (15.2)	0.546
Severe (≥5)	22 (7.6)	4 (8.9)	6 (7.1)	12 (7.6)	
GNRI score, mean ± SD	98.98 ± 8.46	85.09 ± 5.45^‡^	95.12 ± 2.17^‡^	105.01 ± 4.49^‡^	<0.001
Type of surgery, *n* (%)					
General	189 (65.6)	29 (64.4)	53 (62.4)	107 (67.7)	
Orthopedic	71 (24.7)	12 (26.7)	23 (27.1)	36 (22.8)	0.931
Thoracic	28 (9.7)	4 (8.9)	9 (10.6)	15 (9.5)	
Postoperative delirium, *n* (%)	49 (17.0)	14 (31.1)	11 (12.9)	24 (15.2)^‡^	0.021
Length of stay (days), med (IQR)	14 (11)	17 (10)	17 (11)	13 (9)^‡^	<0.001

† *P*‐values according to ANOVA, Kruskal‐Wallis or chi‐squared tests.

‡ Significantly different from the other groups by *post‐hoc* comparison.

CCI, Charlson Comorbidity Index; GNRI, Geriatric Nutritional Risk Index; IQR, interquartile range.

In the univariate logistic analyses, age, type of surgery, CCI and GNRI were significantly associated with POD (Table 2). These four variables were then included in the multivariable logistic regression analysis. After multivariable adjustment, the odds of having POD among patients with a severe/moderate risk of malnutrition were 2.56 times as much as that among those with no nutritional risk (Table 2).

**Table 2:** Univariate and multivariate logistic regression analysis of risk factors for postoperative delirium

Variables	Unadjusted OR (95% CI)	*P*‐value	Adjusted OR (95% CI)	*P*‐value
Age	1.07 (1.00–1.14)	0.04	1.08 (1.01–1.16)	0.028
Male	0.89 (0.48–1.65)	0.711	—	—
Alcohol abuse	0.83 (0.38–1.82)	0.641	—	—
Smoker	1.22 (0.63–2.36)	0.556	—	—
Hearing impairment	1.26 (0.68–2.33)	0.465	—	—
Vision impairment	1.39 (0.72–2.70)	0.325	—	—
Type of surgery				
General	Reference		Reference	
Orthopedic	0.39 (0.16–0.98)	0.044	0.55 (0.21–1.45)	0.225
Thoracic	1.42 (0.56–3.59)	0.463	1.78 (0.65–4.84)	0.261
CCI				
Mild (≤2)	Reference		Reference	
Moderate (3, 4)	3.21 (1.54–6.69)	0.002	3.21 (1.45–7.08)	0.004
Severe (≥5)	6.60 (2.58–16.90)	<0.001	6.35 (2.33–17.33)	<0.001
GNRI				
No risk (>98)	Reference		Reference	
Low risk (92–98)	0.83 (0.39–1.79)	0.634	0.71 (0.32–1.61)	0.418
Severe/moderate risk (<92)	2.52 (1.17–5.43)	0.018	2.56 (1.11–5.89)	0.027

CCI, Charlson Comorbidity Index; CI, confidence interval; GNRI, Geriatric Nutritional Risk Index; OR, odds ratio.

In a linear regression where age, sex, type of surgery and CCI were adjusted, nutritional status was significantly associated with LOS among older surgical patients. On average, patients with a low and severe/moderate nutritional risk stayed in hospital 4.56 and 3.70 days longer than those with no nutritional risk were, respectively (Table 3).

**Table 3:** Linear regression of independent predictors of length of hospital stay

Variable	Model 1		Model 2	
	β (95% CI)	*P*‐value	β (95% CI)	*P*‐value
Age	0.01 (−0.16 to 0.18)	0.916	0.10 (−0.07 to 0.27)	0.242
Male	3.10 (0.95–5.25)	0.005	3.60 (1.43–5.77)	0.001
GNRI				
No risk (>98)	Reference		Reference	
Low risk (92–98)	4.93 (2.47–7.38)	<0.001	4.56 (2.18–6.94)	<0.001
Severe/moderate risk (<92)	3.91 (0.84–6.99)	0.013	3.70 (0.74–6.65)	0.015
CCI				
Mild (≤2)			Reference	
Moderate (3–4)	—	—	2.43 (−0.37 to 5.22)	0.089
Severe (≥5)			2.57 (−1.40 to 6.53)	0.203
Type of surgery				
General			Reference	
Orthopedic	—	—	2.76 (0.15–5.37)	0.039
Thoracic			8.47 (4.83–12.12)	<0.001

Model 1: adjusted for age, sex; Model 2: adjusted for age, sex, CCI and type of surgery.

CCI, Charlson Comorbidity Index; CI, confidence interval; GNRI, Geriatric Nutritional Risk Index.

## Discussion

To the best of our knowledge, this is the first study to explore whether poor nutrition, screened by the GNRI, can predict POD and prolonged LOS in older surgical patients. The results of this prospective cohort study demonstrated that GNRI is a predictive factor for developing POD and prolonged LOS among older patients undergoing non‐cardiac surgery, with lower preoperative GNRI values in these patients being correlated with a poor postoperative prognosis. Our finding suggests that early assessment of nutritional status may help identify those patients who are at risk of POD and prolonged LOS. Furthermore, this finding also raises the question of whether treatment for malnutrition might mitigate the risk of developing POD and longer LOS in at‐risk patients.

In the present study, the prevalence of low and severe/moderate nutritional risk was 45.1%, which was consistent with a previous study that analyzed the impact of GNRI on short‐term mortality in acutely hospitalized older adults.^19^ Dasgupta *et al*. performed a meta‐analysis of 25 publications assessing delirium incidence after non‐cardiac surgery, and they found that the incidence of POD was 5.1%–52.2%.^20^ Our study identified a relatively lower incidence of POD (17%). This difference could be explained by differences in the type of surgery and variations between study populations. Furthermore, the majority of patients in our study underwent elective surgeries.

The GNRI is a promising tool created to study and to predict nutrition‐related complications in the elderly.^10^ It was developed in response to the fact that elderly patients are often unable to participate in questionnaire‐based assessments, and to overcome previous biases linked to the study of unintended weight loss.^1^, ^10^ In addition, it is less time‐consuming and necessitates minimal participation by patients. The GNRI needs only objective parameters that can be readily collected and does not depend on a caregiver or memory. Therefore, it may be useful in elderly patients who have cognitive impairment or delirium.

The National Institute of Clinical Excellence in the UK has advocated for the assessment of nutritional status as a means of delirium prevention. Recently, two studies have found that preoperative malnutrition, evaluated by the MNA‐SF, contributed to the development of POD in orthopedic older patients.^21^, ^22^ However, MNA‐SF or MNA is not applicable to patients with cognitive impairment or those requiring parenteral or enteral nutrition. Compared with the MNA, the GNRI has a poor tendency to classify patients as being at risk or malnourished but appears to predict outcomes better.^11^ To date, only one study has found the association between GNRI and delirium did not reach statistical significance in the coronary intensive care unit, in contrast to our study.^23^ The reason for this inconsistency is likely because the incidence of delirium (9%), as measured by the Intensive Care Delirium Screening Checklist, is somewhat lower than that observed in our study (17%).

There is some evidence that, as a nutritional marker, albumin may be a key risk factor for POD in surgical patients.^24^, ^25^ Nevertheless, albumin levels are more frequently monitored in malnourished patients who are at risk of developing delirium. In addition, it can also be influenced by inflammatory processes, hydration, hepatic impairment and renal impairment. Therefore, it is necessary to combine albumin and a more stable parameter such as body weight, as is the case in the GNRI. We consider that it might be a better indicator than albumin alone. Taken together, these findings support the use of the GNRI as a nutrition‐related factor to predict POD. Even so, more studies are necessary to verify the validity of the GNRI in these patients.

Malnutrition is common in geriatric patients and is often underdiagnosed and indistinguishable from changes associated with the aging process. To date, the relationship between malnutrition and delirium remains unclear. In fact, malnutrition can not only adversely affect the clinical condition of patients, it may increase the risk of poor outcomes such as muscle weakness, impaired immune function, depression, functional impairment and cognitive impairment,^25^, ^26^, ^27^ all of which are risk factors of POD. In addition, the brain is an organ with high metabolic activity and has a high nutritional requirement. Therefore, malnutrition may therefore play an important role in the development of cognitive impairment or delirium. We hypothesize that nutritional supplements can improve the prognosis of surgical patients. Indeed, a previous study found that such nutritional support could reduce the incidence and shorten the duration of delirium.^10^ Based on these findings, we propose that the early detection of malnutrition and subsequent nutritional treatment could reduce rates of postoperative adverse outcomes such as POD.

The association between malnutrition and LOS is well‐established. Until now, the data regarding the association between GNRI and LOS is still lacking. Several studies have investigated the association between GNRI and LOS in older patients. One previous study explored the potential of the NRI as a means of predicting LOS among older patients, and found that NRI independently predicted longer LOS.^28^ Likewise, another study also suggested that malnutrition, as assessed using the GNRI, contributed to in‐hospital weight loss and LOS in elderly patients.^29^ Furthermore, Gartner *et al*. found that GNRI scores correlated with indicators of inflammation and LOS in older inpatients.^30^ The results of our present study are consistent with these previous findings. Our results confirmed that the GNRI is a useful tool for predicting prolonged LOS and that it provided additional insight in non‐cardiac surgical settings.

The study had several strengths. First, the study design was prospective. Moreover, it used a standardized and validated diagnostic instrument (CAM) for the assessment of delirium. Furthermore, the results may be considered representative of a general population of elderly patients due to the fact that this study included various types of surgery. However, this study had several limitations. First, it was a single‐center study with a relatively small sample size (only 288 cases were included). Multicenter and larger studies are required in the future. Second, patients were screened for delirium during the week after surgery owing to practical constraints of our resources. Third, we did not assess the severity or the duration of delirium, and therefore further studies are required to investigate the correlation between malnutrition and the severity or duration of delirium. Finally, because of realistic constraints of our resources, intraoperative data, which might affect the outcomes were not collected in the study.

In the present study, preoperative malnutrition screened by the GNRI was significantly associated with the development of POD and longer LOS in older non‐cardiac surgical patients. We concluded that the GNRI is a useful tool for predicting POD and longer LOS in elderly patients undergoing non‐cardiac surgery. Therefore, using the tool to assess patient nutritional status may help to identify patients more quickly who are at high risk of adverse outcomes, and allowing for early intervention with nutritional supplements, potentially helping to reduce the risk of developing POD and prolonged LOS.

## Disclosure statement

The authors declare no conflict of interest.
